# A single left fourth intercostal thoracotomy approach for resolution of idiopathic chylothorax with thoracic duct ligation and pericardiectomy: a preliminary clinical study in two dogs

**DOI:** 10.3389/fvets.2024.1463939

**Published:** 2024-12-04

**Authors:** Anya K. Price, Kyle G. Mathews

**Affiliations:** Department of Clinical Sciences, College of Veterinary Medicine, North Carolina State University, Raleigh, NC, United States

**Keywords:** thoracic duct ligation, pericardiectomy, idiopathic chylothorax, fourth intercostal space, pleural effusion

## Abstract

Open surgical treatment of idiopathic chylothorax via thoracic duct ligation and pericardiectomy requires a lengthy procedure with two thoracotomy incisions. The objectives of this report were to describe an approach for thoracic duct ligation and pericardiectomy via a single thoracotomy at the left fourth intercostal space and to describe the clinical outcome in two dogs with idiopathic chylothorax. Dogs were prospectively enrolled in a pilot study to evaluate the clinical efficacy of thoracic duct ligation at the left fourth intercostal space, combined with subphrenic pericardiectomy performed through the same incision. Dogs had a preoperative CT lymphangiogram to evaluate the anatomy of the thoracic duct and its branching pattern prior to surgery. Recheck radiographs were performed every 2–4 weeks until effusion resolved. Pleural effusion became non-chylous by 5 days postoperatively. Pleural effusion volume decreased by day 5 postoperatively, allowing removal of thoracostomy tube and discharge from the hospital. Radiographically, effusion resolved within 6 weeks without a need for further drainage after discharge. Dogs remained symptom-free at last follow up (>11 months postoperatively). CT lymphangiograms were repeated >11 months postoperatively and revealed no recurrence of pleural effusion. No intraoperative or postoperative complications directly related to surgery were noted for either dog. Collateral lymphatic vessels were not identified on recheck CT lymphangiograms. The left fourth intercostal approach to thoracic duct ligation and pericardiectomy has potential to be a safe and effective alternative to an open approach requiring two lateral thoracotomies. Further investigation of this approach using open or minimally invasive techniques is warranted.

## Introduction

The etiology of canine idiopathic chylothorax is not well understood, and a variety of surgical interventions are reported. A commonly described surgical treatment of idiopathic chylothorax is ligation of the thoracic duct (TD) to decrease lymphatic flow into the thorax, combined with subphrenic pericardiectomy (1–3). The success rate following thoracic duct ligation and pericardiectomy is reported to be 60–100% for open and 85–95% for thoracoscopic procedures (1–8). Failure of TD ligation is presumed to be due to missed branches precluding complete ligation, or “sleeping vessels,” nearby collateral branches that experience an increase in flow after ligation of identifiable branches (9, 10). The traditional approach for TD ligation, whether open or thoracoscopic, is around the right 8–9th intercostal space, despite reports of multiple branches at this location (11–13). Multiple branches increase the risk for recurrent chylous effusion from missed collateral vessels; therefore, reliable access to a single branch of the TD may result in a higher surgical success rate.

As the canine TD runs cranially from the abdomen into the thorax, it crosses midline from right to left around the level of the fifth intercostal space (14). The cranial TD has been reported to have fewer branches at the left fourth intercostal space than at the traditional right caudal ligation location (14, 15). Ligation of the TD at a location with fewer branches would theoretically decrease the risk of missing a collateral branch and may lead to a higher surgical success rate.

An additional advantage of performing TD ligation at the left fourth intercostal space is pericardial access through the same incision. Pericardiectomy is frequently performed in addition to TD ligation to potentially reduce central venous pressures that may impair the flow of chyle into the venous system (1–3). When performed as an open surgery, the canine TD is approached at the right ninth intercostal space, and the pericardium is approached via a second thoracotomy at the left 5^th^–6^th^ intercostal space. When performed thoracoscopically, an intraoperative change in recumbency is required to perform TD ligation in sternal recumbency and pericardiectomy in dorsal recumbency (6). An additional paracostal incision to inject a mesenteric lymph node with methylene blue for TD visualization may be necessary as well (7, 16, 17). A left fourth intercostal approach would avoid the need for a second thoracotomy in an open procedure and TD intraoperative change in recumbency in a thoracoscopic procedure. If the cranial TD can be visualized with methylene blue injected into a mesenteric lymph node as previously described for contrast lymphangiograms, a paracostal abdominal incision would be unnecessary, further decreasing surgical morbidity (16, 17).

A left fourth intercostal approach to TD ligation and pericardiectomy has been described in normal canine cadavers and was successful in stopping the flow of contrast in the cranial mediastinum (15). This approach has not yet been reported for treatment of idiopathic chylothorax in clinically affected dogs. The objective of this manuscript was to describe this alternative surgical approach to the thoracic duct and pericardium at the fourth intercostal space, describe the perioperative findings, and to evaluate the clinical outcomes of dogs surgically treated via this approach.

## Materials and methods

Dogs diagnosed with idiopathic chylothorax at the North Carolina State Veterinary Hospital were prospectively enrolled in a clinical trial from January 2022 to June 2024. Owners were given the option to enroll their dog into this study or have a standard right-sided thoracoscopic TD ligation performed. A consent form was signed prior to entry into this pilot study. The diagnosis of idiopathic chylothorax was given to dogs with a chylous pleural effusion determined by paired serum and fluid triglyceride levels and no clear underlying cause as determined by bloodwork (complete blood count, chemistry profile, Idexx 4DX SNAP**®** test to screen for tick borne disease), echocardiogram, or thoracic radiographs. A computed tomographic (CT) lymphangiogram was performed to characterize the anatomy of the thoracic duct. Dogs were excluded if they had a disease suspected to be contributing to the chylothorax (e.g., lung lobe torsion or cardiomyopathy). During the study period, five dogs with chylothorax presented to the North Carolina State Veterinary Hospital, and two dogs were enrolled. One dog was diagnosed with atrial fibrillation and was not enrolled. Two dogs were treated with thoracoscopic duct ligation at the right caudal thorax due to owner preference for a minimally invasive procedure. Signalment, history, physical exam findings, diagnostic imaging results, surgical reports, and outcome, including follow-up thoracic radiographs and CT lymphangiogram, were recorded. A successful outcome was defined as resolution of pleural effusion postoperatively within the short-term follow-up period (6 weeks) with no need for additional surgical intervention. Mortality was defined as death or euthanasia during the perioperative period or directly related to signs associated with chylothorax. The present study was approved by the Institutional Animal Care and Use Committee at North Carolina State University (No. 22-327).

### Case 1

A 2 year, 11 month old female spayed Goldendoodle was referred for idiopathic chylothorax. The dog was initially evaluated by its primary veterinarian 3 months prior to referral for a decreased appetite, vomiting, coughing, and an increased respiratory effort. Thoracic radiographs identified pleural effusion, and the dog was referred to a specialty hospital for further workup. At this visit, the dog had a complete blood count, chemistry profile, and a urinalysis performed which revealed no significant abnormalities aside from elevated alanine aminotransferase (ALT). Point of care ultrasound (POCUS) of the thorax and abdomen revealed severe bilateral pleural effusion with no B-lines, and no significant findings in the abdomen. Thoracic radiographs revealed severe pleural effusion as well as a patchy alveolar-interstitial pattern. A thoracocentesis was performed, and approximately 1,500 mL of pleural fluid was removed. Cytology of the fluid revealed a lipid-rich effusion (triglycerides 620 mg/dL compared to 63 mg/dL in peripheral blood) as well as variably atypical and hyperplastic mesothelial cells, suspected to be reactive mesothelial cells with dysplasia in response to the chylous effusion. An Idexx 4DX SNAP**®** test was performed to screen for tick-borne disease, and was negative. An echocardiogram was performed to look for evidence of cardiomyopathy, neoplasia, or arrhythmias that could be contributing to chylothorax, and found no evidence of underlying cardiovascular disease. A CT lymphangiogram was performed at another specialty hospital and revealed no evidence of structural disease or underlying causes of chylothorax, consistent with a diagnosis of idiopathic chylothorax. The dog was prescribed a low fat diet (Royal Canin GI low fat) and rutin (62 mg/kg q8h) for medical management of a chylothorax. The dog required repeat thoracocentesis twice within 3 months, which led owners to seek surgical treatment.

**Figure 1:**
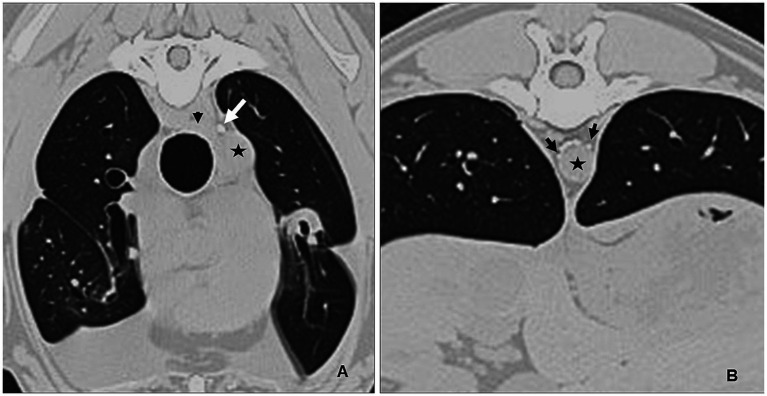
Preoperative CT lymphangiogram transverse images Case #1. **(A)** Level of the fourth intercostal space. A single left sided thoracic duct (white arrow) is present lateral to the esophagus (black arrowhead) and dorsal to the descending aorta (star). **(B)** Caudal thorax. Multiple thoracic duct branches are present (between black arrows) dorsal to the aorta (star). Left is to the right of the images.

On presentation to the North Carolina State Veterinary Hospital, the dog was bright, alert and responsive and had a normal respiratory rate. Decreased heart and lung sounds were auscultated ventrally. Abnormalities noted on complete blood count and chemistry profile included a mild thrombocytopenia (137 × 10^3^/μL, reference range 190–468), moderate lymphopenia (0.343 × 10^3^/μL, reference range 0.59–3.3), and mildly elevated ALT (95 IU/L, reference range 17–78). Given that a previous CT lymphangiogram had been conducted without evidence of contrast extravasation, a subsequent CT lymphangiogram was deemed necessary. This follow-up procedure employed contrast injection into the carpal pads to enhance visualization and characterization of the cranial thoracic duct anatomy while also aiming to identify any cranial extravasation of contrast in the cranial mediastinum that may have been previously undetected. The decision to utilize carpal pad injection was made in an effort to improve the diagnostic yield and provide more comprehensive imaging of the lymphatic system in the cranial region. The lymphangiogram was performed by injecting 11 mL of 300 mgI/mL iohexol (Omnipaque™) into each carpal pad (22 mL total, 1 mL/kg). The dose of 1 mL/kg was chosen as previous studies have successfully visualized the thoracic duct on CT lymphangiogram with 0.75 mL/kg of iohexol (18). Thoracic CT sequences were run at 5, 10, and 15 min post-injection and identified mild–moderate bilateral pleural effusion, cranial mediastinal lymphadenopathy (likely reactive), and mild pneumothorax, suspected to be due to recent thoracocentesis. On the original CT, one well highlighted branch of the TD was noted at the planned surgical site (fourth intercostal space), and 2–5 branches of the thoracic duct were noted at the standard surgical site (T8-T10 on the right), with variability at this site due to branching and re-joining (Figure 1). Numerous tortuous lymphatics were noted in the cranial mediastinum but the terminus of those vessels could not be identified. No additional information was gained from the forelimb lymphangiogram. No extravasation or leakage of contrast medium into the pleural space was identified on either CT.

#### Surgery

**Figure 2:**
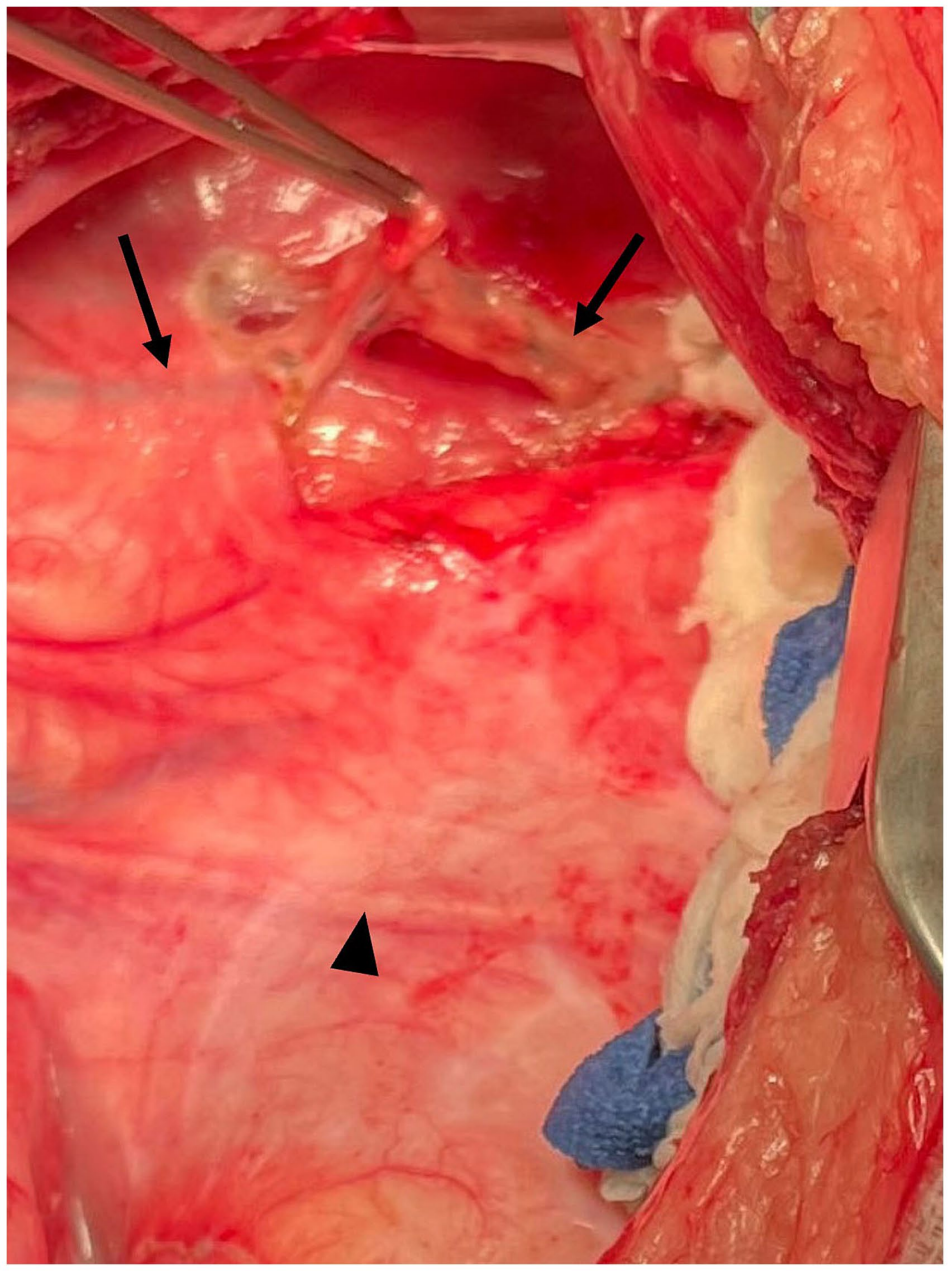
Intraoperative photograph Case #1. Following dissection through the mediastinum dorsal to the aorta, the thoracic duct (arrows) is easily elevated from the lateral surface of the esophagus. Mediastinal thickening was more pronounced in this case. The arrowhead indicates the phrenic nerve. Cranial is to the left of the image, dorsal is to the top of the image.

While under anesthesia prior to surgery, a mesenteric lymph node was identified on abdominal ultrasound and 0.6 mL of methylene blue (0.3 mg/kg) was injected into the lymph node. The dog was then moved into the operating room (OR) and placed in right lateral recumbency. An approximately 15 cm skin incision was made at the left fourth intercostal space and a routine approach was made into the pleural space. Approximately 500 mL of chylous effusion was suctioned from the thoracic cavity. The left cranial lung lobe was normal in appearance and was packed caudally with moist laparotomy sponges. Moderate thickening of the mediastinum and pericardial surface was evident. The TD was visualized after initial removal of the thick mediastinal surface just dorsal to the aorta, between the left subclavian artery and the first visible intercostal artery. The duct (stained blue) was easily observed as a single branch on the lateral surface of the esophagus. The thoracic duct was elevated and dissected using right-angle forceps (Figure 2). Two medium-large hemoclips were placed across the duct caudally, and one medium-large hemoclip was placed approximately 2 cm cranial to the caudal clips. The portion of the TD in between the two hemoclips was then transected by sealing the section with a harmonic scalpel (Johnson & Johnson, New Brunswick, NJ). The esophagus was retracted ventrally so that the dorsal and right surfaces could be inspected. No other lymphatic branches were identified.

The pericardium was easily visualized and accessed through the left fourth intercostal approach. The pericardium was substantially thickened. Using the harmonic scalpel, a subtotal pericardiectomy was performed to remove as much pericardium as possible ventral to the phrenic nerve. The removed pericardium was submitted for histopathology. A 14 g thoracostomy tube (Mila International, Florence, KY) was placed percutaneously. Closure was routine. Procedure time was 92 min.

### Case 2

A 4 year, 2 month old male neutered mixed breed dog (26.7 kg) was referred for surgical treatment for idiopathic chylothorax. The dog initially presented to its primary veterinarian for a routine wellness visit. Owners had not noted exercise intolerance, but had noticed recent weight loss and a decreased appetite. On auscultation, heart sounds were decreased, so thoracic radiographs were performed. Pleural effusion was noted and the dog was referred to the Emergency Department at the North Carolina State Veterinary Hospital. On presentation, the dog had an elevated respiratory rate at 100 bpm, an elevated heart rate at 130 bpm, and an elevated temperature at 103.2 F. Heart and lung sounds were muffled on auscultation. A thoracocentesis was performed, removing 2,400 mL of pleural fluid, and fluid analysis revealed a lymphocyte-rich fluid with a triglyceride content of 771 mg/dL. No bacterial growth was observed on aerobic or anaerobic culture. Bloodwork (complete blood count and chemistry profile) revealed mildly elevated lactate of 3.6 mmol/L (reference range 0–3), mature neutrophilia (segmented neutrophils 9.47 × 10^3^/μL, reference range 2.84–9.11), moderate lymphopenia (0.37 × 10^3^/μL, reference range 0.59–3.3), mild thrombocytopenia (160 × 10^3^/μL, reference range 190–468), moderate hypophosphatemia (1.6 mg/dL, reference range 2.6–5.3), mild hypocholesterolemia (119 mg/dL, reference range 151–348), and a mild hypokalemia (3.5 mmol/L, reference range 3.6–5.3). An Idexx 4DX SNAP**®** test was performed to screen for tick-borne disease and was negative. An echocardiogram found no evidence of underlying cardiovascular disease. The dog was discharged with a low fat diet and treatment with rutin (50 mg/kg q8 h). The dog needed multiple thoracocenteses over the course of the next 2 months. To better characterize the thoracic duct anatomy, a CT lymphangiogram was performed at the North Carolina State Veterinary Hospital by injecting 14 mL of 350 mg[I]/mL iohexol into each metatarsal footpad (28 mL total, 1 mL/kg) followed by 5 min of limb massage. Thoracic CT sequences were run at 5, 10, and 15 min post-injection. An additional 7 mL of contrast was injected per metatarsal footpad (0.5 mL/kg) with an additional scan performed after 5 min as contrast did not reach the area of interest. Following this, the carpal footpads were injected with 14 mL iohexol per footpad (1 mL/kg) and following 5 min of massage scans were performed at 5 and 10 min post injection attempting to better visualize the cranial thoracic duct. The total volume of contrast administered was 2.5 mL/kg. Despite administration of additional contrast, TD branches cranial to the diaphragm were difficult to identify. One branch was noted at the planned surgical site (fourth intercostal space), and 1–2 branches were noted at the standard surgical site (T8-T10 on the right). The terminus of the TD could not be identified. No extravasation or leakage of contrast medium into the pleural space was identified. A moderate volume of bilateral pleural effusion was present. Moderate sternal and mild cranial lymphadenopathy was noted, with mild intra-abdominal lymphadenomegaly and mild right ventriculomegaly. The dog was scheduled for surgery for TD ligation and pericardiectomy.

#### Surgery

Prior to induction, a left-sided thoracentesis was performed and 1,300 mL of milky/pink cloudy fluid was aspirated. Prior to moving into the OR, abdominal ultrasound was used to identify a mesenteric lymph node and 5 mL of 1% methylene blue (1.8 mg/kg) was injected into the node. The dog was moved into the OR and placed in right lateral recumbency. An approximately 15 cm skin incision was made at the left fourth intercostal space. A total of 1,500 mL of chylous effusion, light green in color from intermixed methylene blue, was suctioned from the thoracic cavity. The left cranial lung lobe appeared normal and was packed caudally with moist laparotomy sponges. Mild to moderate thickening of the mediastinum and pericardial surface was evident.

**Figure 3:**
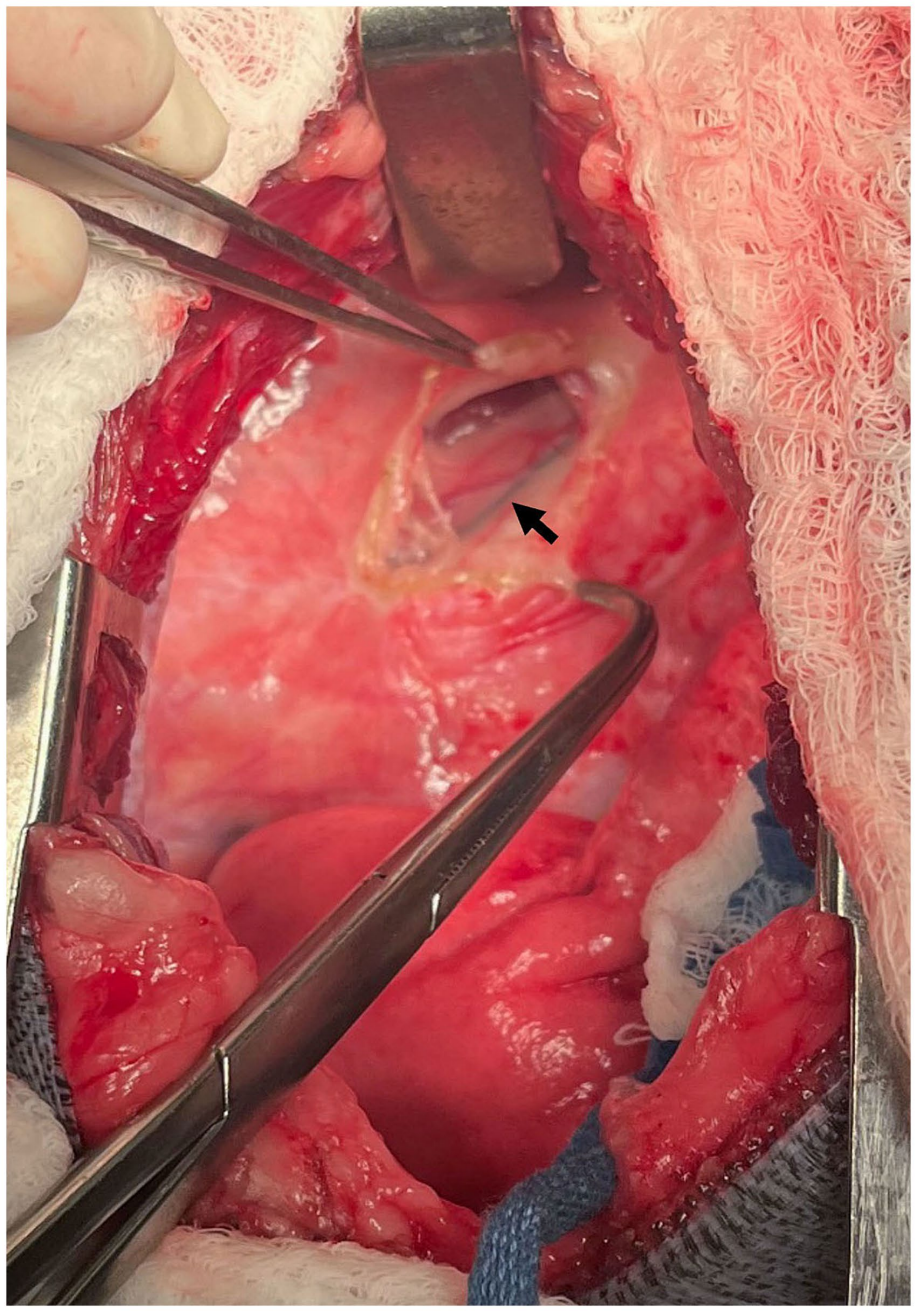
Intraoperative photograph at the left fourth intercostal space. Case #2: The methylene blue-stained thoracic duct can be seen (arrow) with the mediastinum on the lateral surface of the esophagus. Cranial is to the left of the image, dorsal is to the top of the image.

**Figure 4:**
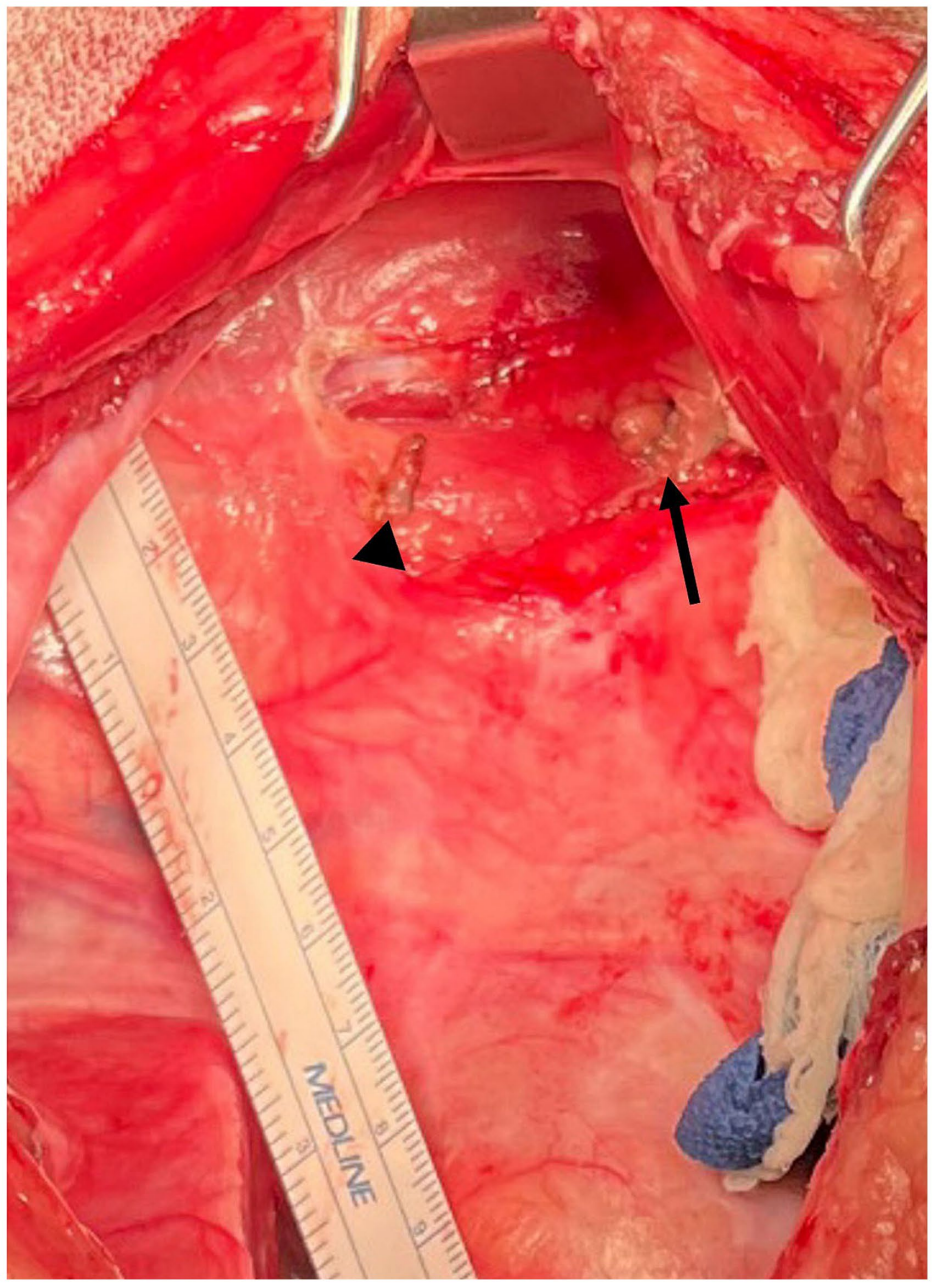
Intraoperative photograph Case #2: The thoracic duct has had clips applied and the intervening portion of the thoracic duct sealed and removed using a harmonic scalpel. The arrowhead indicates the cranial clip and the arrow indicates the caudal clips. Cranial is to the left of the image, dorsal is to the top of the image.

The TD was easily visualized due to the methylene blue uptake, lying lateral to the esophagus (Figure 3). Several small serpentine lymphatics running in a dorsoventral direction were also stained in the cranial mediastinum. The TD was isolated with right angle forceps. One medium-large hemoclip was placed across the duct cranially and two medium-large hemoclips were placed caudally, approximately 2 cm from the cranial clip. The portion of the TD in between the hemoclips was then sealed and transected with the harmonic scalpel (Figure 4). The esophagus was retracted ventrally so that the right surface could be inspected. No other lymphatic branches were identified on the right side of the esophagus.

**Figure 5:**
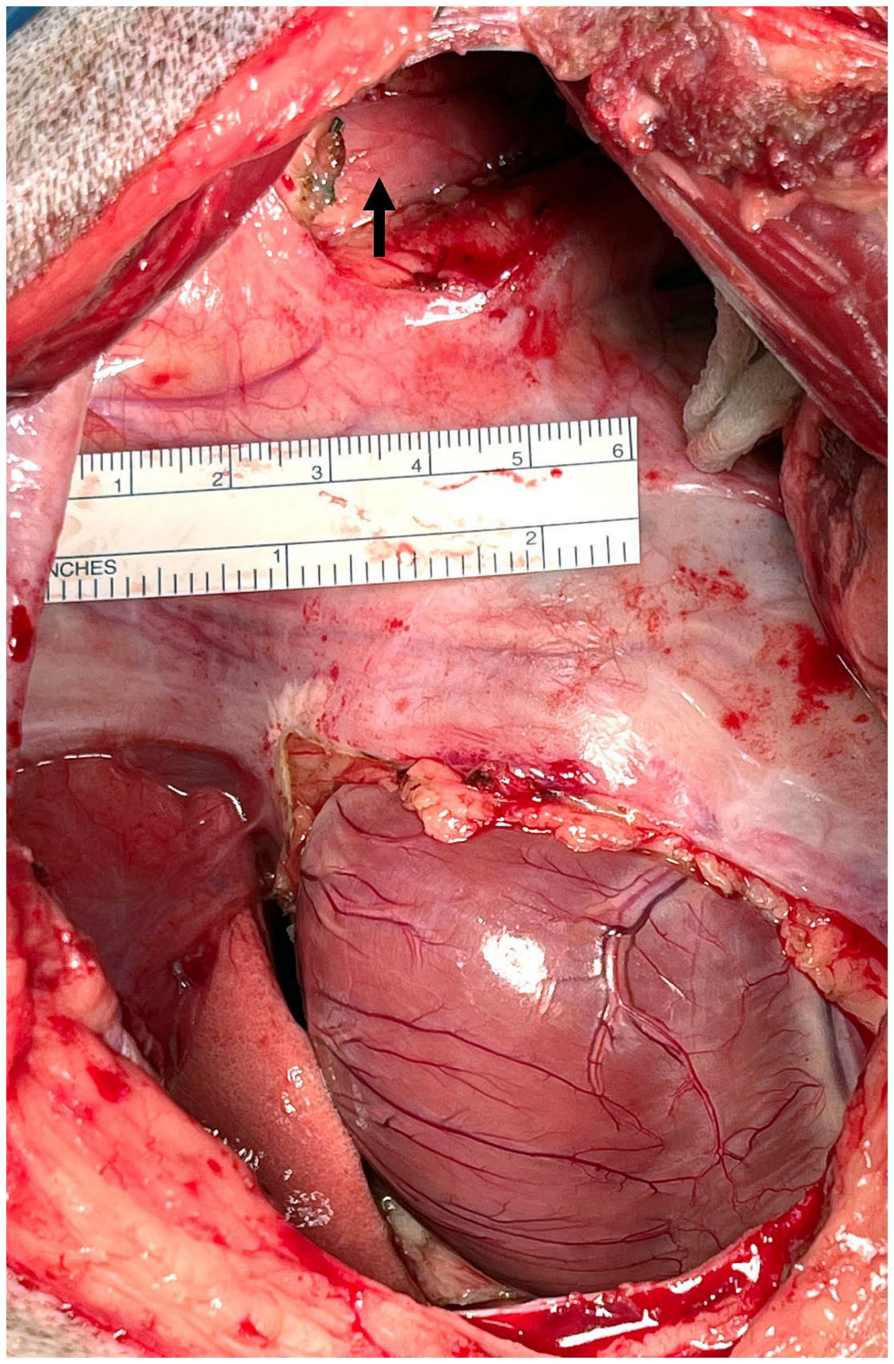
Intraoperative photograph Case #2: Subphrenic pericardiectomy was performed to remove as much pericardium as possible through access via the left intercostal thoracotomy. The arrow indicates the ligated, sealed and transected thoracic duct. Cranial is to the left of the image, dorsal is to the top of the image.

The pericardium was easily visualized and accessed through the left fourth intercostal approach. A subphrenic pericardiectomy was performed with the harmonic scalpel, excising as much tissue as possible ventral to the phrenic nerve (Figure 5). The pericardium was removed and submitted for histopathology. A 14 g thoracostomy tube was placed percutaneously in the left hemithorax. Closure was routine. Procedure time was 62 min.

## Results

### Case 1

The dog recovered from anesthesia without complication and was monitored in the intensive care unit (ICU). The thoracostomy tube was aspirated per standard protocol, starting with aspiration every 1 h and decreasing frequency between aspirations depending on the level of air and fluid obtained. Overnight, no fluid was aspirated from the thoracostomy tube but a large volume of air was aspirated (1,029 mL). The thoracostomy tube was aspirated from then on only as needed out of concern for an iatrogenic pneumothorax, in order to allow a leak to seal itself. Two days postoperatively, effusion triglycerides were 258 mg/dL. Three days postoperatively, effusion triglycerides decreased to 65 mg/dL, lower than serum triglycerides (100 mg/dL). Three days postoperatively the tube produced 120 mL fluid (6.3 mL/kg) and 40 mL of air over the course of 20 h. The chest tube was removed and the dog was discharged 3 days post-operatively. Histopathology of the pericardium revealed minimal, multifocal, acute, neutrophilic pericarditis, with no overt evidence of infectious agents or neoplasia. The diagnosis of pericarditis was consistent with the thickened, fibrous appearance of the pericardium in surgery.

Thoracic radiographs were performed 2 weeks postoperatively and revealed resolution of pleural effusion. Thoracic radiographs were taken by the primary veterinarian 3 months postoperatively and revealed no recurrence of pleural effusion. Follow-up was obtained via email 287 days after discharge and the dog was reportedly doing well with no concerns from the owner.

**Figure 6:**
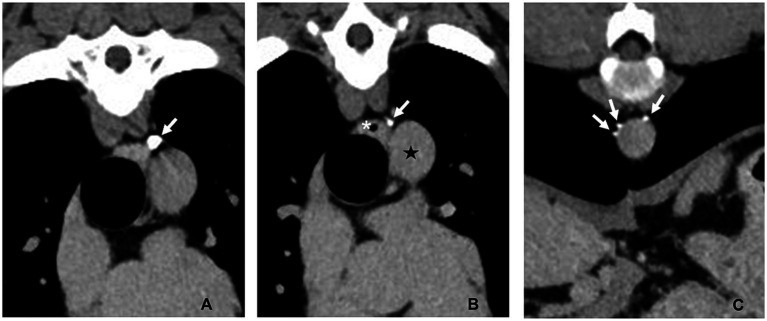
CT lymphangiogram transverse images of Case #1 13 months postoperatively. **(A)** Fourth intercostal space. The caudal most hemoclip (arrow) is lateral to the esophagus and dorsal to the aorta. **(B)** Fourth intercostal space 3 mm caudal to the image in panel **(A)**. A single thoracic duct branch (arrow) can be seen lateral to the esophagus (asterisk) and dorsal to the descending aorta (star). **(C)** Ninth intercostal space. Three thoracic duct branches (arrows) can be seen dorsal and right lateral to the aorta. Left is to the right of the images.

**Figure 7:**
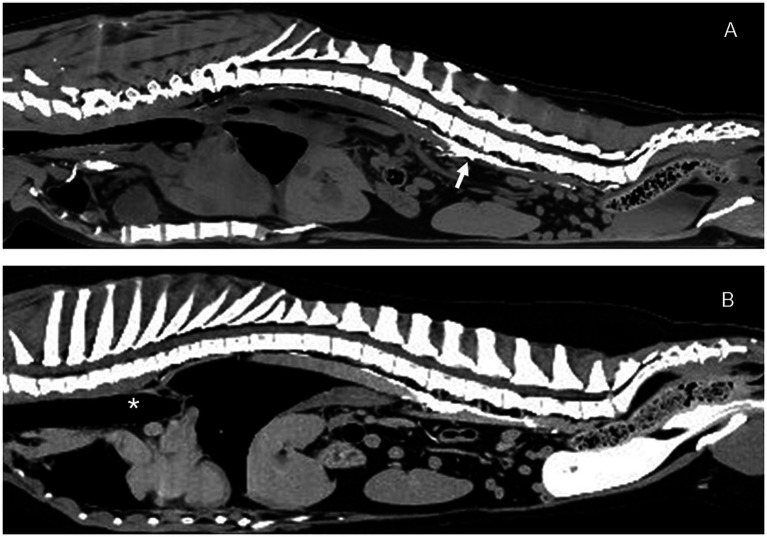
Sagittal CT lymphangiogram images of Case # 1. **(A)** Preoperative image showing contrast in the region of the cisterna chyli (arrow). **(B)** 13 month postoperative image showing relatively unchanged abdominal lymphatics. No additional lymphatic collaterals were noted. Thoracic duct ligation site (asterisk). Cranial is to the left of the images.

The dog was evaluated for a recheck CT lymphangiogram 13 months postoperatively to document any changes to thoracic duct morphology. The dog had reportedly been doing very well since the last appointment and had no episodes of respiratory distress. No abnormalities were detected on physical exam. A CT lymphangiogram was performed under general anesthesia by injecting 12 mL of 300 mg[I]/mL of iohexol into each metatarsal footpad (24 mL total; 1 mL/kg), followed by 5 min of limb massage. Thoracic CT sequences were run at 5, 10, and 15 min post-injection. The TD was easily identified with good contrast uptake stopping at the level of the previously placed caudal hemoclips. No leakage of contrast was noted, and no changes to the lymphatic system, including collateral lymphatics, were identified (Figures 6, 7). The pleural effusion had resolved, with only scant fluid noted around the plica vena cava.

### Case 2

The dog recovered from anesthesia without complication and was monitored in the ICU. The thoracostomy tube was aspirated per standard protocol, starting with aspiration every 1 h and increasing frequency between aspirations depending on the level of air and fluid obtained. One day post-operatively, point-of-care ultrasound (POCUS) of the thorax revealed a moderate volume of persistent pleural effusion, and a total of 610.9 mL of fluid was drained from the thoracostomy tube over 24 h (23.6 mL/kg/day). A sample of the pleural effusion was submitted for triglycerides, which were 141 mg/dL. Two days post-operatively, POCUS of the thorax revealed mild pleural effusion on the right hemithorax, and an identical total of 610 mL of fluid was drained from the thoracostomy tube over 24 h (23.6 mL/kg/day). Triglycerides of the effusion 2 days post-operatively were 410 mg/dL. Over the next 3 days, the dog’s pleural fluid became progressively more serosanguinous in appearance. Fluid production was 13.4 mL/kg/day by day 3 postop and 23.9 mL/kg/day by day 4 post-op. By day 5 post-operatively, fluid production had decreased to 8 mL/kg/day, and the thoracostomy tube was pulled. Triglyceride levels 5 days post-operatively were normal at 48 mg/dL (last reference of serum triglycerides was from 5 days previously; 84 mg/dL). Histopathology of the pericardium revealed normal tissue, with no evidence of inflammation or neoplasia, despite the gross appearance of mild to moderate thickening of the pleura and pericardium identified in surgery.

Thoracic radiographs were performed 2 weeks postoperatively and identified persistent mild to moderate pleural effusion that was markedly improved from prior imaging. Thoracic radiographs were repeated at 6 weeks postoperatively and revealed further improvement, with scant pleural effusion. The owner was contacted for follow-up via email 180 days after discharge, and reported that the dog was doing well 10 months postoperatively.

The dog was evaluated 11 months postoperatively, and had reportedly been doing very well since the last appointment, with an improved appetite since surgery and no episodes of respiratory distress. No abnormalities were detected on physical exam. A CT lymphangiogram was performed under general anesthesia by injecting 14 mL of 340 mg[I]/mL iohexol into each metatarsal pad (28 mL total; 1 mL/kg), followed by 5 min of limb massage. Transverse thoracoabdominal CT sequences were acquired at 5, 10, and 15 min post-injection. This was followed by a second 7 mL injection each metatarsal pad due to lack of contrast reaching the site of ligation. Uptake of contrast was not noted cranial to T13; caudal to that, no leakage of contrast was noted, and no changes to the lymphatic system including development of collateral lymphatics within the abdomen were identified.

## Discussion

This pilot study describes the successful clinical outcome of two dogs with idiopathic chylothorax after TD ligation and pericardiectomy with a left fourth intercostal approach. The TD was easily identified after methylene blue lymph node injection and was accessible with minimal dissection. Chylous pleural effusion and clinical signs resolved in both dogs within 6 weeks postoperatively. Pleural effusion became non-chylous by 5 days postoperatively in both dogs and decreased significantly in volume by day 5 postoperatively to allow for removal of thoracostomy tube and discharge from the hospital. Pleural effusion resolved by 6 weeks in both dogs without need for additional thoracocentesis. Both dogs remained symptom free at >11 months postoperatively. Neither dog had intraoperative or postoperative complications related to the surgery. No lameness was noted in either dog after preoperative or recheck CT lymphangiogram from injection of contrast in the tarsal or carpal pads, and no significant residual swelling was noted in the areas of injection, likely due to vigorous and extensive massage that was performed immediately prior to the lymphangiogram.

This study also demonstrated the success of ultrasound-guided injection of lymph nodes with methylene blue for visualization in surgery. While ultrasound-guided mesenteric lymph node injection has been previously described, this study demonstrated the efficacy of methylene blue injection into the mesenteric lymph node reaching the cranial thoracic duct (16, 17). The mesenteric lymph nodes were easily accessed and injected by a board-certified radiologist. One of the dogs was inadvertently administered 5 mL (1.8 mL/kg) of methylene blue instead of the desired 0.5–1.0 mg/kg, but no adverse effects were noted as a result of this error. The successful visualization of the cranial TD after ultrasound-guided injection of a mesenteric lymph node eliminates the need for an abdominal incision to access a mesenteric lymph node, decreasing surgical time and surgical morbidity. The time of ultrasound injection to the time of surgical incision was not recorded for Case 1, but the TD remained highlighted for the entire procedure (92 min). The time of ultrasound injection to the time of surgical incision was 41 min in Case 2, and the thoracic duct remained highlighted for the entire procedure (62 min). Procedure times listed include closure times, so it is possible that the TD could not be visualized for an entire 92 min of surgery once obscured by other structures. In the event that appropriate visualization of the TD was not achieved, the dog could be draped widely to include the lateral abdomen to allow a second intraoperative ultrasound-guided injection to be performed, or allow a small paracostal incision to be made to access a mesenteric lymph node.

Thoracic duct ligation has historically been performed on the TD in the right caudal mediastinum (1–3). Due to the unknown etiology of idiopathic chylothorax, concern exists that a more cranial ligation may bypass a leak or anomaly of the vessel. However, contrast lymphangiograms of dogs with idiopathic chylothorax reveal that dogs with identifiable lymphatic abnormalities have these abnormalities (such as dilated lymphatics) in the cranial mediastinum, cranial to the left fourth intercostal space (12, 13, 16, 17, 19). Therefore, ligation of the TD caudal to these abnormalities would theoretically decrease the flow of lymph through anomalous lymphatics. The left fourth intercostal approach was found to be successful in this pilot study, adding support to this theory. Follow up of both dogs >11 months after surgery revealed no concerns for recurrent pleural effusion, and no changes to the lymphatic system that could be identified on CT lymphangiogram.

When applied in a minimally invasive setting, use of a thoracoscopic cranial left-sided approach with a dog in right lateral recumbency may be a less invasive way and more efficient surgical treatment. This approach may allow access to fewer branches of the TD as well as the pericardium through the same portals, while avoiding an intraoperative recumbency change needed to perform a TD ligation (in sternal recumbency) and pericardiectomy (in dorsal recumbency) thoracoscopically, leading to shorter anesthesia and surgery times.

Limitations of this report include the small sample size. Successful outcome was judged on lack of clinical signs of pleural effusion and radiographic resolution of pleural effusion. Right atrial pressures were not measured before and after pericardiectomy, so the need for pericardiectomy is unclear in these cases. Histopathologic analysis of the pericardium revealed pericarditis in Case #1, consistent with the grossly thickened appearance, and a normal pericardium in Case #2, which was inconsistent with the mildly thickened appearance of the pleura and pericardium. While pericardiectomy may not be necessary for treatment in dogs with idiopathic chylothorax and no evidence of constrictive pericardial physiology (10), this approach nevertheless demonstrates an approach for reliable access to the TD, with convenient access to the pericardium in the event that a pericardiectomy is indicated.

Individualized surgical planning and pre-operative imaging is important for optimal management of idiopathic chylothorax. While a cranial left-sided approach led to successful outcomes in the two dogs in the present study, an atypical cranial right-sided branch or an unusual leak of chyle in the mid-to-caudal mediastinum would likely not lead to successful outcomes with this approach. CT lymphangiography is strongly advised prior to surgery to understand the branching pattern of the TD. CT lymphangiography using iohexol injection into the metatarsal pads led to reliable surgical findings in these two dogs although contrast uptake in the TD of Case #2 was weak on both pre- and postoperative CTs. Additional CT images following carpal pad injection added no useful information in these cases.

In conclusion, this pilot study provides evidence to support use of a left fourth intercostal approach for combined TD ligation and pericardiectomy for treatment of idiopathic chylothorax in dogs. This technique, combined with ultrasound-guided injection of methylene blue for TD visualization, may reduce morbidity in dogs by eliminating the need for a second thoracotomy and the need for an abdominal incision. Given the success of the procedure, a larger prospective study is advised to gain a better appreciation of outcomes with this approach.

## Data Availability

The raw data supporting the conclusions of this article will be made available by the authors, without undue reservation.

## References

[1] Fossum TW Mertens MM Miller MW Peacock JT Saunders A Gordon S. Thoracic duct ligation and pericardectomy for treatment of idiopathic chylothorax. J Vet Intern Med. (2004) 18:307–10. doi: 10.1111/j.1939-1676.2004.tb02550.x, PMID: 15188816

[2] Carobbi B White RA Romanelli G. Treatment of idiopathic chylothorax in 14 dogs by ligation of the thoracic duct and partial pericardiectomy. Vet Rec. (2008) 2008:743–5. doi: 10.1136/vr.163.25.743 19103616

[3] Reeves LA Anderson KM Luther JK Torres BT. Treatment of idiopathic chylothorax in dogs and cats: a systematic review. Vet Surg. (2020) 49:70–9. doi: 10.1111/vsu.13322, PMID: 31508821

[4] McAnulty JF. Prospective comparison of cisterna chyli ablation to pericardectomy for treatment of spontaneously occurring idiopathic chylothorax in the dog. Vet Surg. (2011) 40:926–34. doi: 10.1111/j.1532-950X.2011.00902.x, PMID: 22091690

[5] Radlinsky MG Mason DE Biller DS Olsen D. Thoracoscopic visualization and ligation of the thoracic duct in dogs. Vet Surg. (2002) 31:138–46. doi: 10.1053/jvet.2002.31062, PMID: 11884959

[6] Allman DA Radlinsky MG Ralph AG Rawlings CA. Thoracoscopic thoracic duct ligation and thoracoscopic pericardectomy for treatment of chylothorax in dogs. Vet Surg. (2010) 39:21–7. doi: 10.1111/j.1532-950X.2009.00623.x, PMID: 20210940

[7] Mayhew PD Steffey MA Fransson BA Johnson EG Singh A Culp WTN. Long-term outcome of video-assisted thoracoscopic thoracic duct ligation and pericardectomy in dogs with chylothorax: a multi-institutional study of 39 cases. Vet Surg. (2019) 48:O112–20. doi: 10.1111/vsu.13113, PMID: 30376180

[8] Kanai H Furuya M Hagiwara K Nukaya A Kondo M Aso T. Efficacy of en bloc thoracic duct ligation in combination with pericardiectomy by video-assisted thoracoscopic surgery for canine idiopathic chylothorax. Vet Surg. (2020) 49:O102–11. doi: 10.1111/vsu.13370, PMID: 31880337

[9] Kanai H Furuya M Yoneji K Hagiwara K Nukaya A Kondo M. Canine idiopathic chylothorax: anatomic characterization of the pre- and postoperative thoracic duct using computed tomography lymphography. Vet Radiol Ultrasound. (2021) 62:429–36. doi: 10.1111/vru.12966, PMID: 33684240

[10] Mayhew PD Balsa IM Stern JA Johnson EG Kaplan J Gonzales C. Resolution, recurrence and chyle redistribution after thoracic duct ligation with or without pericardiectomy in dogs with naturally occurring idiopathic chylothorax. J Am Vet Med Assoc. (2023) 261:696–704. doi: 10.2460/javma.22.08.0381, PMID: 36563067

[11] Birchard SJ Cantwell HD Bright RM. Lymphangiography and ligation of the canine thoracic duct: a study in normal dogs and three dogs with chylothorax. J Am Anim Hosp Assoc. (1982) 18:769–77.

[12] Singh A Brisson B Nykamp S. Idiopathic chylothorax: pathophysiology, diagnosis, and thoracic duct imaging. Compend Contin Educ Vet. (2012) 34:E1–8. 22935990

[13] Iwanaga T Tokunaga S Momoi Y. Thoracic duct lymphography by subcutaneous contrast agent injection in a dog with chylothorax. Open Vet J. (2016) 6:238–41. doi: 10.4314/ovj.v6i3.13, PMID: 27995081 PMC5155138

[14] Korpes K Kolenc M Trbojević Vukičević T Đuras M. Anatomical variations of the thoracic duct in the dog. Anat Histol Embryol. (2021) 50:1015–25. doi: 10.1111/ahe.12745, PMID: 34632615

[15] Price AK Mathews KG Lawver JE Scharf VF. Evaluation of thoracic duct ligation and unilateral subphrenic pericardiectomy via a left fourth intercostal approach in normal canine cadavers. Vet Surg. (2024) 53:437–46. doi: 10.1111/vsu.14060, PMID: 38078621

[16] Johnson EG Wisner ER Kyles A Koehler C Marks SL. Computed tomographic lymphography of the thoracic duct by mesenteric lymph node injection. Vet Surg. (2009) 38:361–7. doi: 10.1111/j.1532-950X.2008.00473.x, PMID: 19573100

[17] Enwiller TM Radlinsky MG Mason DE Roush JK. Popliteal and mesenteric lymph node injection with methylene blue for coloration of the thoracic duct in dogs. Vet Surg. (2003) 32:359–64. doi: 10.1053/jvet.2003.50044 12865998

[18] Kim K Cheon S Kang K Hwang Y Oh D Yoon J. Computed tomographic lymphangiography of the thoracic duct by subcutaneous iohexol injection into the metatarsal region. Vet Surg. (2020) 49:180–6. doi: 10.1111/vsu.13324, PMID: 31576584

[19] Fossum TW Birchard SJ. Lymphangiographic evaluation of experimentally induced chylothorax after ligation of the cranial vena cava in dogs. Am J Vet Res. (1986) 47:967–71. PMID: 3963604

